# Sweat Testing and Recent Advances

**DOI:** 10.3389/fped.2021.649904

**Published:** 2021-05-04

**Authors:** Yasemin Gokdemir, Bulent Taner Karadag

**Affiliations:** Department of Pediatric Pulmonology, Marmara University, Istanbul, Turkey

**Keywords:** cystic fibrosis, diagnosis, sweat test, Gibson-Cooke, chloride

## Abstract

Cystic fibrosis (CF) is the most common fatal genetic disease of the Caucasian population. Sweat testing is the principal diagnostic test for CF, and it is used for the evaluation of infants with positive CF newborn screening (NBS) and in patients with clinical findings suggesting CF. This article describes the classical sweat test method in detail and also provides an overwiew of recent advances.

## Introduction

High concentrations of chloride (Cl^−^) detected in sweat from patients in the early 1940s resulted in the development of the sweat Cl^−^ test (ST), and by 1959, the test was being used by Gibson and Cooke (1). Since the discovery of the cystic fibrosis (CF) gene, encoding the CF transmembrane regulator (CFTR) protein in 1989, more than 2000 mutations have been reported. The CFTR is located on the apical membrane of the epithelial cells in the exocrine secretory system that includes the sweat glands. Defective CFTR function mainly results in abnormal Cl^−^ transport across the Cl^−^ channels as well as diminished sodium transport across the cell membrane. Reduced Cl^−^ secretion and enhanced sodium reabsorption across the epithelial cells increases the viscosity of secretions, and in the sweat, Cl^−^ concentration is elevated (1–3).

Detection of elevated values of sweat Cl^−^ by the quantitative pilocarpine iontophoresis test (QPIT) performed via chloridometer is accepted as the gold standard in CF diagnosis. This technique is performed in three stages: cholinergic stimulation of sweating with iontophoresis, collection of the sweat sample, and measurement of sweat Cl^−^ concentration (4–9).

Well-organized and accurate ST procedures are especially important in countries with limited access to genetic testing. After the introduction of new CFTR modulators in the treatment of CF, ST has become even more important. Besides its role in the diagnosis of CF, normalization of sweat Cl^−^ concentrations after administration of modulator therapies is used as proof of their efficacy (10, 11).

Because QPIT is relatively complicated to carry out; the technique and the multiple steps of the process need to be well-understood (6). Several evidence-based guidelines on how to perform the test properly have been published; each including a detailed description about sweat induction, collection, analysis, and interpretation (4–6, 8, 9). The English language guidelines were developed by the Clinical and Laboratory Standards Institute (CLSI, USA), Multi-Disciplinary Working Group (UK), and The Australasian Association of Clinical Biochemists (AACB, Australasia) (4–6). National guidelines are also available in French and Turkish and are actively used in France and Turkey (8, 9).

The CLSI guideline for ST was revised in 2019 and is officially recommended by the American Cystic Fibrosis Foundation (CFF) (4). According to this guideline, there is a single agreed-upon methodology for pilocarpine iontophoresis and sweat collection but several acceptable methods for sweat analysis (7). Recent reports, however, state that there are still differences in laboratory techniques employed in testing in many countries, particularly lower income countries. Poor adherence to published guidelines suggests an inability to meet quality standards in laboratory diagnostics and, consequently, casts doubt on the accuracy of the results (10–12).

Lack of access to pilocarpine, equipment, and trained laboratory staff, coupled with the relative difficulty of the recommended technique for QIPT have caused a search for an ST method that is easier to carry out. Additionally, collecting a sufficient amount of sweat can be challenging in infants (13, 14). For these reasons, some devices that use different, simpler methods have been developed for measuring sweat Cl^−^, and the safety and efficacy of these methods are reported by controlled studies (15–20). In this study, we review the QIPT and the newer alternatives now being employed in the diagnosis of CF.

## Indications

The ST is indicated for individuals suspected to have CF, either from positive NBS or the presentation of clinical features suggestive of CF. CF genotyping is recommended in all patients with positive or borderline ST results and also in patients in whom ST is not technically possible. It is also necessary to identify which CF patients are eligible for CF-mutation-specific therapy. The CFF recommends CF genotyping in all patients diagnosed with CF (7, 11, 12, 21).

Patients with CF may have a variety of clinical manifestations. Some neonates may have meconium ileus but have IRT levels in the normal range; in these cases, ST should be carried out. Young children may have pulmonary complications, such as recurrent pneumonia, chronic sinusitis, nasal polyps, or persistent and recurrent wheezing and coughing. The most common gastrointestinal findings are failure to thrive with malabsorptive stools and recurrent abdominal pain. Patients with these findings should be referred to the ST center (7, 21–23).

Because sweat Cl^−^ concentration can be temporarily elevated in the first day of life, ST should be done later than 48 h after birth and optimally at the 10th day (4). ST should be postponed in premature infants until they reach 2 kg of weight and more than 36 weeks corrected gestational age. Ideally, the child should be well-hydrated and should not have acute illness. ST can be carried out for subjects requiring oxygen via mask or nasal canula. Newborns and infants that are receiving open system oxygen in the incubator should not be tested because sparks can be produced during the iontophoresis phase of the sweat test when a low electric current is applied (4, 22, 23).

## Sweat Collection

It is recommended to perform ST in an accredited care center by a trained technician (4–7). The ST is typically performed on the patient's arm or leg. The test starts with iontophoresis of pilocarpine, a parasympathomimetic alkaloid, which acts on the cholinergic receptors by mimicking acetylcholine, to stimulate sweat production by sweat glands. Collection of two simultaneous samples is recommended because of the variability of the test and insufficient sample risk (4).

In the original Gibson and Cooke method, iontophoresis is done by placing two electrodes on the patient's arm or leg and covering one of them with pilocarpine-soaked gauze and the other with deionized water–soaked gauze. An electric current of maximum 1.5 mA is then applied for 5 min to stimulate sweat production. The electrical stimulation is painless and causes no discomfort. Sweat is collected for a period of up to 30 min. For the gauze or filter paper method, the stimulated area must be 2 × 2 inches. The filter paper is then placed in a laboratory dish of known weight so that the quantity of the collected sweat can be calculated. The minimum quantity required for sweat collected from the gauze method is 75 mg (4). However, conventional procedures, such as those using gauze and filter paper, carry a significant risk of evaporation unless performed by trained and experienced staff. Errors made during sweat stimulation and collection and analysis can cause skin burns and also volumetric, gravimetric, condensate, and evaporation inaccuracies. This is especially significant in young, particularly preterm, infants (4, 13).

In 1983, the Wescor (Logan, Utah) Macroduct system of sweat collection was developed. This technique was easier to perform than the conventional QIPT and requires only 15 ml of sweat. Gel discs containing pilocarpine are utilized, and the iontophoretic current passing through these discs stimulates sweat production. For safety, the iontophoretic current source needs to be battery powered. Capillary tubing is used for collecting the sweat produced by induction of sweat glands.

The sample is analyzed using a variety of techniques. In young infants, however, there is still a higher risk of insufficient quantity (QNS) ST. McColley et al. reports that 27% of CF Centers in the United States state a mean QNS rate of 10.5% in infants 14 days old or younger (13, 24, 25). Collecting sweat from two sites is, therefore, recommended in infants. Bilateral testing increases the likelihood of collecting a sufficient specimen from at least one site. If the quantity of sweat collected from one site is insufficient for analysis, the sweat samples from various sites should not be combined; in this case, the ST should be repeated (26, 27).

**Figure 1:**
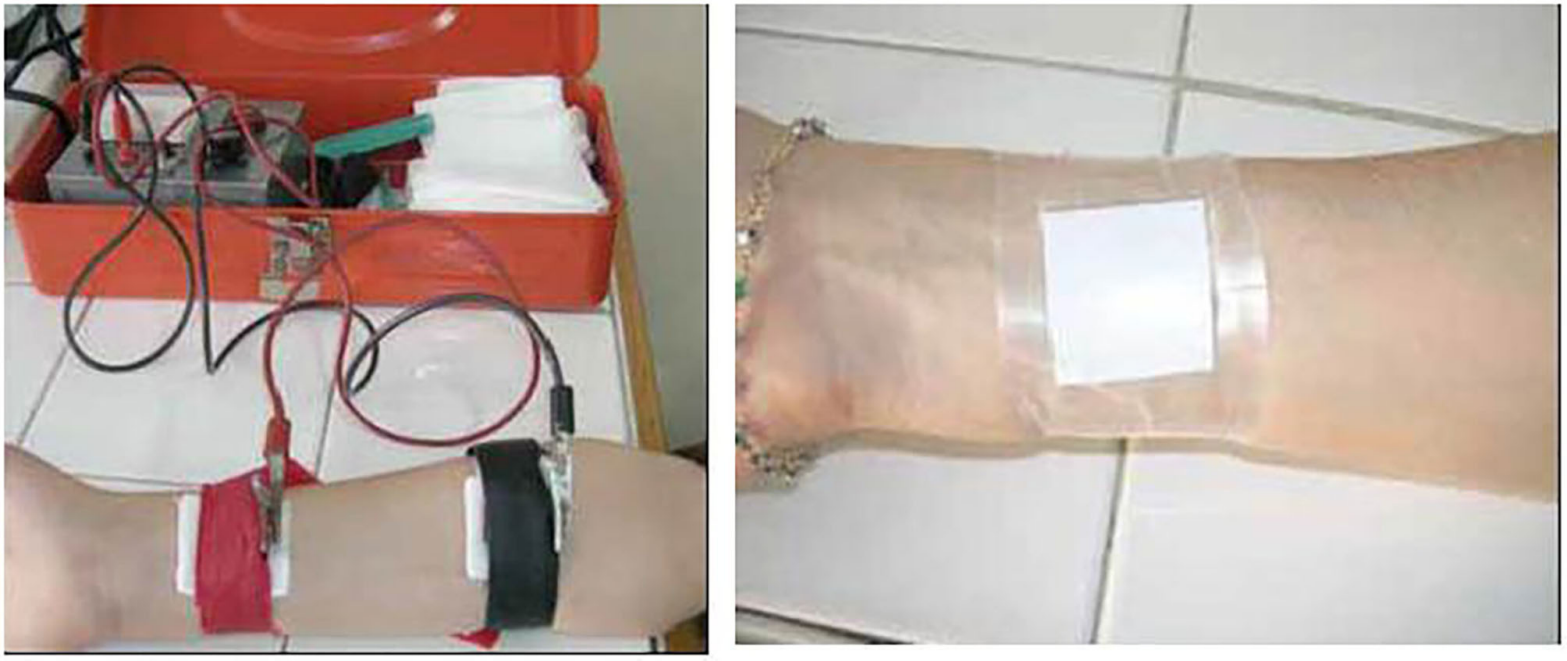
Iontopheresis and sweat collection by Gibson-Cooke method.

**Figure 2:**
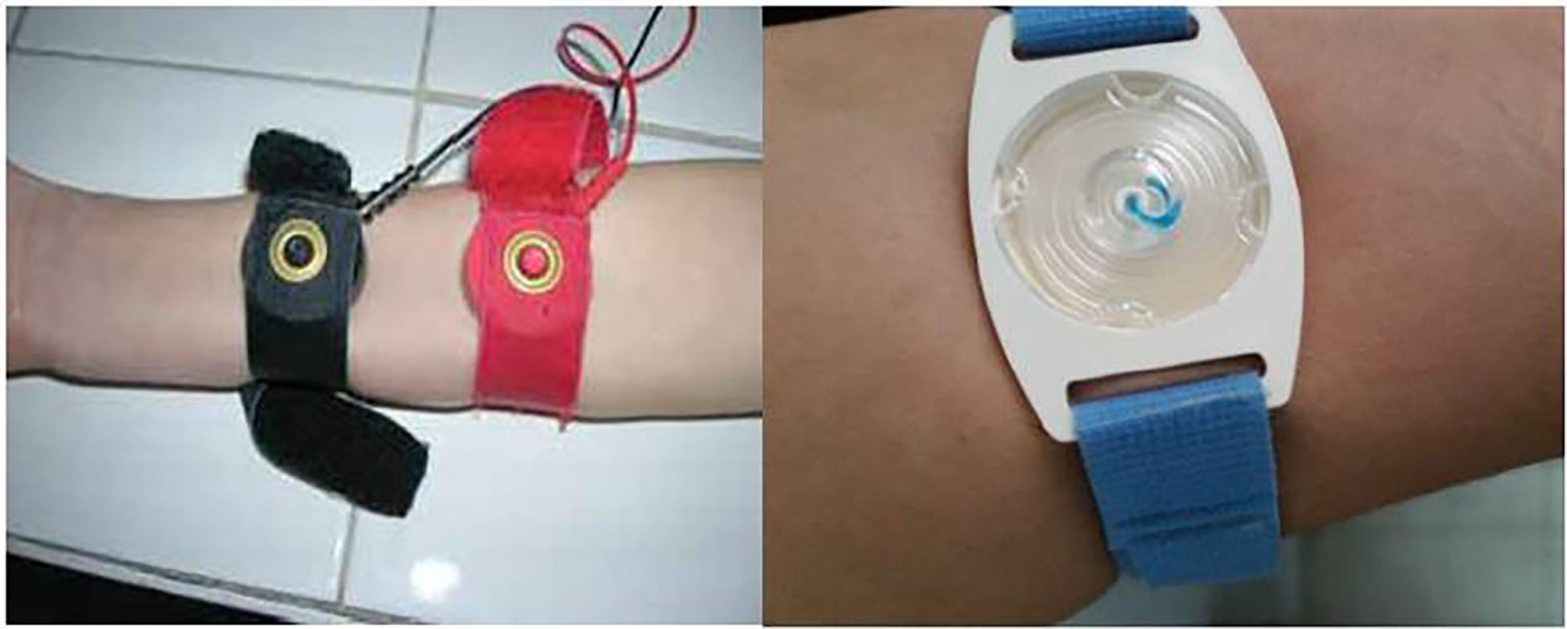
Iontopheresis and sweat collection by Macroduct System.

Currently, both the Gibson and Cooke QPIT and the Macroduct^®^ systems are recommended for sweat collection in CF diagnosis (Figures 1, 2). The Chloridometer, the conventional device for sweat Cl^−^ analysis, also utilizes the Macroduct^®^ coil for sweat collection (4).

In studies comparing these two systems, equivalent results are reported. The only significant difference between the Gibson and Cooke and Macroduct^®^ QPIT sweat collection systems was sample matrix. The sample for the Gibson and Cooke QPIT is diluted because of the need for elution from the collection medium. The Macroduct^®^ QPIT sample is collected into tubing and can be analyzed directly.

## Biochemical Analysis

Coulometry is the unique method of sweat Cl^−^ analysis approved and described in detail in the CLSI guidelines; it involves coulometric titration with a chloridometer. A chloridometer measures the free Cl^−^ concentration in an acidic solution by allowing current to flow through a circuit. Free Cl^−^ ions bind with silver cations generated from silver electrodes to form silver Cl^−^ molecules; these no longer conduct the electrical current. The chloridometer measures how much current flows and how long it flows for in order to determine how many free Cl^−^ ions are in the solution at the beginning of the process. Using a specimen volume of ≥15 μL, a chloridometer can measure Cl^−^ concentrations from 10 to 160 mmol/L (4).

Sweat collection and analysis should be performed on the same day, and the results and their interpretation should be reported to clinicians as soon as possible. Analysis of the sweat shortly after collection or within a few hours should be routine procedure for the ST center (21). Collected sweat should not be stored or transported via the coiled tubing system because of the evaporation risk. If a significant delay is expected between collection and analysis, the laboratory may store specimens in 0.2-mL microcentrifuge tubes for 72 h without significant evaporation (4). For the purpose of shipment, in the case of collection of sweat during clinical trials, the specimens can be stored frozen (−70°C) and accurately analyzed later.

The ion-selective electrode (ISE) method may also be used to measure Cl^−^, but there are limited data available on its use. This method converts the activity of a specific ion dissolved in a solution into an electrical potential, which is measured by a voltmeter (3). The ISE technique is included in the UK and Australian ST guidelines as an acceptable method; however, it has not been validated systematically for accuracy of sweat Cl^−^ measurements. The CLSI suggests that, if it is used, the laboratory must validate the method against the traditional ST. The major concern about ISE measurement is that decreased sensitivity at lower concentrations could lessen the precision of the result (4–6).

## Sweat CL^–^ Interpretation

**Figure 3:**
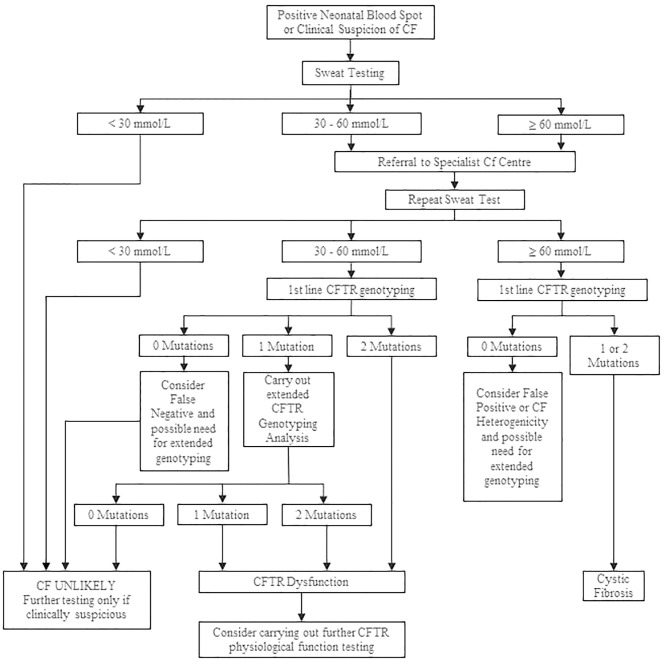
Cystic fibrosis diagnostic algorithm. Adapted from Simmonds (12).

The cutoff value for sweat Cl^−^ testing is the same regardless of a patient's age (7, 28). A level of Cl^−^ of higher than 60 mmol/L in the sweat is indicative of CF; concentrations lower than 30 mmol/L are considered normal, and CF is unlikely. A level between 30 and 59 mmol/L is defined as intermediate (borderline), and repeated ST or additional diagnostic tests are required (28). After a positive ST result, either ST is repeated or genetic testing is performed to confirm the definite CF diagnosis (12). A diagnostic algorithm for CF for interpreting the results of the ST is presented in Figure 3.

## Interfering Factors

Variability in sweat Cl^−^ levels is shown in previous studies, but sweat Cl^−^ biological variability, in both healthy people and CF patients is not well-known (29, 30). Results from a total of 5,960 tests from two CF centers were reported by Vermeulen et al. According to this study, in 90% of subjects, −3.2 and +3.6 mmol/L changes were obtained from samples taken on both sides collected at the same test occasion. However, two separate tests showed much higher variability with changes between −18 and +14 mmol/L in 90% of the subjects. Biological variability mostly affected the intermediate test results, and some of them returned to within normal range with the repeated tests in that study. On the other hand, sweat Cl^−^ values higher than 60 mmol/L showed small biological variability (30).

Another concern regarding ST is false positive cases. The most common reason for a false positive ST is technical error during the procedure, such as evaporation of the sweat sample. The incidence of this problem is reduced by correct implementation and adherence to recommended testing procedures and by ensuring that the test is performed in adequately equipped laboratories and by properly trained personnel (4).

Sweat Cl^−^ levels may also be elevated falsely in other pathologic conditions, including atopic dermatitis, ectodermal dysplasia, pseudohypoaldosteronism, untreated hypothyroidism, glycogen storage disease type I, carbonic anhydrase XII mutations, malnutrition, and anorexia nervosa. Elevated sweat Cl^−^ concentrations in non-CF patients may also be related to iatrogenic causes, such as mineralocorticoid, NaCl^−^ perfusion, and topiramate treatment (21). The underlying mechanism for false positive results in many conditions is unknown. The possible sweat gland function impairment associated with the skin manifestations may be the reason for high levels of sweat Cl^−^ in patients with atopic dermatitis and ectodermal dysplasia (31). Hyperchlorhidrosis caused by otosomal recessive inherited Carbonic Anhydrase XII deficiency should be considered in the differential diagnosis of a positive ST, especially with the clinical findings of hyponatremic dehydration during infancy. High sweat Cl^−^ levels during treatment with topiramate may be the result of the inhibition of carbonic anhydrase isotypes in the sweat gland ducts (32).

## Reporting Results

Name, surname, and the date of birth of the patient and the date and hour of the test should be recorded. The type of ST employed; the level of Cl^−^ measured; the unit of measurement; if the value is normal, borderline, or high; and the interpretation of the test result should all be specified in the test report. Cl^−^ concentrations in whole numbers should be reported using mmol/L units. In quantitative Cl^−^ measurements, mmol/L and milliequivalent per liter (mEq/L) are equivalent. It is not necessary to report the total sweat volume collected if an adequate volume was ensured (4).

## Other ST Methods

### Conductivity

Although quantitative sweat Cl^−^ measurement is the unique approved method for CF diagnosis, sweat conductivity measurement is easier and also commonly used in many settings throughout the world. Conductivity depends on the concentration and mobility of the ions within a solution and reflects a non-selective measurement of ions. As the bicarbonate and lactate ions in the sweat affect the conductivity, results do not show the sweat Cl^−^ concentration (26, 27, 33). Mean sweat conductivity test results are ~15–20 mmol/L higher than sweat Cl^−^ measurement. Several sweat conductivity measuring instruments are available.

A conductivity instrument was approved as a screening method (8). A sweat conductivity test result is defined as abnormal if the value is ≥50 mmol/L, and these patients should be referred for a quantitative ST to confirm the diagnosis of CF (4).

According to the CLSI guideline, conductivity should not be used as a diagnostic test for infants with a positive NBS result. These babies should be tested with quantitative ST (4). There are many studies, however, that have compared the conductivity ST method with conventional coulometric ST and that show adequate efficacy and safety (15, 33, 34).

Nanoduct^®^ is one of the ST devices used with the conductivity method. It was developed especially for use with newborns, and only 3 μL of sweat is required. As soon as sweat enters the microconductivity cell, the ST result is shown on the display (35).

In the national NBS program for CF in Switzerland, Macroduct^®^ collection (with Cl^−^ concentration measurement) and Nanoduct^®^ test (measuring conductivity) methods were compared. Although only 60% of Macroduct^®^ tests were successful at the first attempt, the Nanoduct^®^ had a higher rate of successful outcome (79%), and it was as sensitive as the Macroduct^®^ in identifying newborns with CF (sensitivity 98 vs. 99%, respectively) but less specific (specificity 79 vs. 93%) (35). Another study from the Netherlands also showed that Nanoduct^®^ fails less often in newborns than the Gibson and Cooke/Macroduct^®^ because it can operate with a small quantity of sweat, and it is, therefore, advocated that it can be used to confirm the diagnosis CF in infants with a positive NBS test for CF (36).

### Coulometric Endpoint Method

The coulometric endpoint method utilizes an electrolysis reaction measuring the changes in resistance to the current flowing between electrodes. The concentration of the solution is equivalent to the current generated. Sweat is collected by a tube similar to Macroduct^®^ coil that reduces the risk of sample evaporation. This method is approved by the CLSI, UK, and Australian ST guidelines (4–6).

The CFΔ Collection System^®^ is a new-generation, ST analyzer manufactured by UTSAT, which is based on this coulometric end point method. Studies comparing this device with quantitative sweat Cl^−^ analysis demonstrate that the coulometric end point method is safe and can be as reliable as gold standard methods. This device is approved by CE, commercially available, and routinely used in Turkey, in some of the Middle East and African countries, and in Azerbaijan (18, 37).

This method was compared with both the titrimetric Cl^−^ measurement (Sherwood^®^ Chloridometer 926S, Sherwood Scientific Ltd., Cambridge, UK) and the classical Gibson and Cooke and manual titration methods. Bland–Altman plots were used to analyze the agreement between methods in the healthy controls and the CF subjects. Good agreement was obtained between the coulometric end point technique and the gold standart ST methods (18).

### Ion Exchange Technology: Wearable Sweat Sensor

This technique is based on ion exchange technology accepted by the CLSI Guidelines and measures quantitative Cl– levels with the accuracy of the traditional method (19).

Recently, several reports have been published on wearable sweat Cl analyzer for CF diagnosis (16, 19). CF Quantum^®^ Sweat Test System (CFQT) is one example that is awaiting approval by the FDA and the CE; it is manufactured by Medtronic Inc., Minneapolis, Minnesota. Sweat production is stimulated with pilocarpine via a portable, wearable electrode and collected by a Cl^−^ test patch. Finally, the sweat Cl^−^ value is calculated by an analyzer after scanning the patch with a camera. The time needed to achieve a reliable result is short, and the result is reported in 30 min with a small sample volume (9 μL). This technique was compared with the conventional coulometric method in both CF patients and healthy controls. The sweat Cl^−^ concentrations obtained from the wearable sensor showed excellent agreement with the conventional tests with a Pearson correlation coefficient of *p* = 0.97. Sweat Cl^−^ measurements for all healthy subjects were within the accepted threshold for normal (≤29 mEq/L; 16–27), and all individuals with CF were above the accepted threshold (≥60 mEq/L; 65–130), confirming CF diagnosis. The correlation coefficient between the CFQT and conventional ST was 0.98 [95% confidence interval (CI): 0.97–0.99]. The sensitivity and specificity of the CFQT in diagnosing CF was 100% (95% CI: 94–100%) and 96% (95% CI: 89–99%), respectively (16).

### Inductively Coupled Plasma Mass Spectrometry (ICP-MS)

ICP-MS is used in the clinical laboratory on a routine basis to an increasing extent, mainly to determine the presence of oligoelements. It can also be employed for sweat Cl^−^ assay and provides accurate measurements, especially at low Cl^−^ concentrations (38). Pullan et al. used ICP-MS to analyze sweat Cl^−^ and sodium for the measurement of ST, collected via a Macroduct^®^ sweat collector tube (39). Collie et al. demonstrated that both online (instrument based) and off-line (sample based) internal standard methods measuring Cl– were successful in providing accurate, reproducible results (17). Marvelli et al. conducted a study comparing this method with gold standard coulometric titration in 50 healthy volunteers and two CF patients. The method was then cross-validated by assaying 50 standard samples with Cl^−^ concentration values in the range 10–131 mM by both ICP-MS and coulometric titration. Bland–Altman plots confirmed the analogous concentration levels for coulometric titration and ICP-MS; bias had a value of −0.9 (95% CI = −1.96 ÷ 0.20) with lower and upper limits of agreement of −8.3 (95% CI = −10.18 ÷ −6.47) and 6.6 (95% CI = 4.71 ÷ 8.42), respectively. Consequently, the authors report good correlation between the two Cl^−^ analysis techniques (38). Although not involved in any of the current English language ST guidelines, ICP-MS is utilized by a number of ST laboratories, especially in Australia, the UK, and Italy. Authors confirm this method is as safe and accurate as the conventional coulometric method and suggest it be recognized as a candidate reference method for the monitoring and diagnosis of CF (17, 39, 40).

### Capillary Electrophoresis: Skin Wipe Test (SWT)

In the SWT, unstimulated, spontaneously formed sweat is collected using a cotton swab moistened with deionized water, then extracted. The collection procedure is non-invasive and faster than the conventional ST method. Evaluation by SWT with contactless conductivity detection, typically performed in the biochemical laboratory, analyzes the whole “sweat ionome” (41).

Durc et al. compared SWT with the conventional coulometric method in 114 CF patients, 76 healthy carriers, and 58 controls. The SWT method with capillary electrophoretic analysis for CF diagnosis performed comparably with the conventional Macroduct^®^ ST. The SWT method evaluated Cl^−^/K^+^ and (Cl^−^+Na^+^)/K^+^ ion ratios for CF diagnosis. Two ion ratios, Cl^−^/K^+^ and (Cl^−^+Na^+^)/K^+^, from the SWT samples and Cl^−^ values from the ST samples were evaluated to diagnose CF. Sensitivity of the SWT method using the Cl^−^/K^+^ ratio (cutoff value 3.9) was 93.9% compared with 99.1% when using the (Cl^−^+Na^+^)/K^+^ ratio (cutoff value 5.0) and 98.3% in using Macroduct Cl^−^ (cutoff value higher or equal to 60 mmol/L). The method specificities were 97.8, 94.0, and 100.0%, respectively. The authors propose the SWT as a new diagnostic technique for CF (20).

### ST for Outcomes in Clinical Trials

The increased use of CFTR modulators in the treatment of CF has highlighted the need for precise and accurate biomarkers to evaluate their efficacy. These therapies may not result in equivalent clinical improvement for all CFTR mutations, and the Cl^−^ concentration in sweat can serve as a useful biomarker of CFTR function, *in vivo*, in assessing the response to modulator treatments (18). In terms of the treatment response, studies show correlations between functional classes of CFTR variants and sweat Cl^−^ concentration (19, 20). In clinical use, baseline and serial sweat Cl^−^ measurements are usually used to monitor the effects of therapies targeting CFTR function in previously diagnosed CF patients (4, 16, 17, 21).

## Conclusion

Sweat Cl^−^ concentration is the first-choice test to confirm a CF diagnosis. In addition to this, it is also essential in monitoring the efficacy of modulator treatments. All steps of ST are subject to a risk of error, resulting from inexperienced laboratory personnel or lack of appropriate quality assurance. Inaccurate methodology of the sweat collection, technical error, and misinterpretation of the results are all possible. Additionally, with the increasing frequency of NBS all over the world, the need for ST in the neonatal period and also in very low-weight babies is increasing. These have all led to efforts to create easier to carry out but still reliable, ST methods and procedures. Although, as described in the paper, a number of newer methods have been developed and are being used, these methods still need careful interpretation in decision making for CF.

## Author Contributions

YG and BK have made contributions to the design, editing, and writing of this manuscript. All authors contributed to the article and approved the submitted version.

## Conflict of Interest

The authors declare that the research was conducted in the absence of any commercial or financial relationships that could be construed as a potential conflict of interest.
